# A reusable mesoporous adsorbent for efficient treatment of hazardous triphenylmethane dye wastewater: RSM-CCD optimization and rapid microwave-assisted regeneration

**DOI:** 10.1038/s41598-021-02213-2

**Published:** 2021-11-23

**Authors:** Payam Arabkhani, Hamedreza Javadian, Arash Asfaram, Seyed Nabiollah Hosseini

**Affiliations:** 1 Department of Chemistry, Tehran North Branch, Islamic Azad University, Tehran, Iran; 2 Chemistry & Chemical Engineering Research Center of Iran (CCERCI), P.O. Box 14335-186, Tehran, Iran; 3 Medicinal Plants Research Center, Yasuj University of Medical Sciences, Yasuj, Iran

**Keywords:** Environmental sciences, Environmental social sciences, Chemistry, Materials science, Mathematics and computing, Nanoscience and technology

## Abstract

In this research, mesoporous calcium aluminate nanostructures (meso-CaAl_2_O_4_) were synthesized using a citric acid-assisted sol–gel auto-combustion process as the potential adsorbent to eliminate toxic triphenylmethane dye malachite green (MG) from synthetic/real effluent. The surface morphology of meso-CaAl_2_O_4_ was highly porous with nanometric size and non-homogeneous surface. The specific surface area, total pore volume, and BJH pore diameter of meso-CaAl_2_O_4_ were 148.5 m^2^ g^−1^, 1.39 cm^3^ g^−1^, and 19 nm, respectively. The meso-CaAl_2_O_4_ also showed a very high heat resistance, due to losing only 7.95% of its weight up to 800 °C, which is mainly related to the moisture loss. The optimal adsorption conditions were obtained based on response surface methods (RSM)-central composite design (CCD) techniques. The Langmuir isotherm model was used for fitting the adsorption measurements, which presented 587.5 mg g^–1^ as the maximum adsorption capacity of the dye. The data obtained from the adsorption kinetics model were found to correspond to the pseudo-second-order model. Also, the thermodynamic parameters including enthalpy change (ΔH°), entropy change (ΔS°), and Gibbs free energy change (ΔG°) indicated that MG dye adsorption by the meso-CaAl_2_O_4_ was feasible, endothermic, and occurred spontaneously. Furthermore, the meso-CaAl_2_O_4_ was regenerated by microwave irradiation under 900 W at 6 min, and the MG dye removal efficiency was remained over 90% after the five cycles of microwave regeneration.

## Introduction

**Figure 1:**
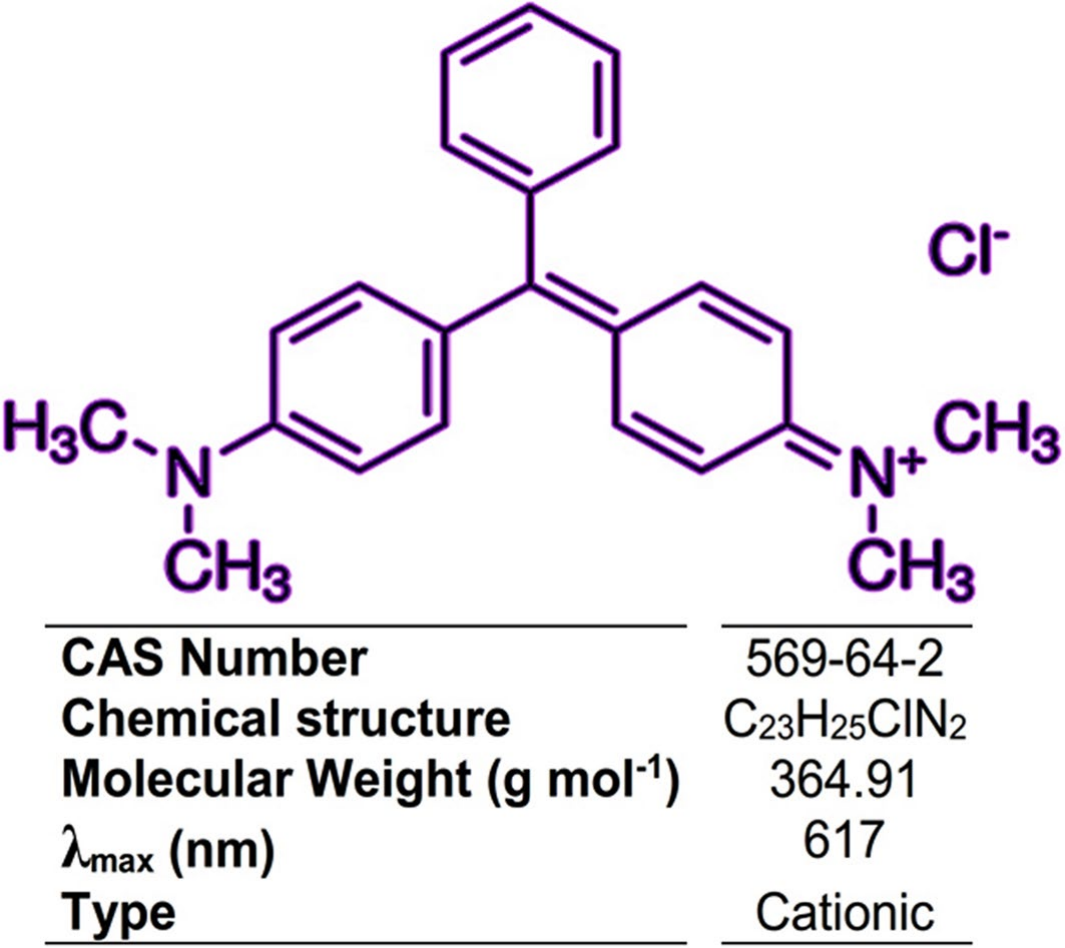
Chemical structure of MG dye.

Water contamination is one of the most serious problems facing the world today. Among various pollotions such as pesticides, pharmaceutical materials, heavy metals, microplastics, and so on, dyes are highly radioactive, non-biodegradable and carcinogenic^1^. Dyes are abundant in the effluents of many manufacturing industries, including food, plastics, cosmetics, and textiles^2^. Consequently, it is critical to eliminate dyes from manufacturing effluents before releasing them into the environment. The textile industry ranks first in dye usage, and textile wastewater treatment is challenging^3, 4^. As a triphenylmethane cationic dye, malachite green (MG, Fig. 1) is one of the most widely used synthetic colorants applied to dye silk, leather, cotton, and wool in textile industries^5^. MG dye is also used in the aquaculture industry due to its high effectiveness as an antibacterial, antifungal, and antiparasitic agent. Although MG dye in food fish production has been banned in many countries, due to its low cost and high efficacy is still used^6^. However, the consumption of MG dye contaminated water is dangerous and carcinogenic due to the presence of nitrogen in its structure and causes severe damage to humans and animals^7^. Therefore, the effective removal of MG dye residue in water and wastewater is still a serious environmental challenge. Various treatments methods such as physicochemical, biochemical, and electrochemical processes have been utilized to remove MG dye from textile wastewater^8^. On the other hand, the resistance of MG dye to light and oxidizing agents causes that biological and chemical deposition to have difficulty for its removal from wastewaters. Adsorption has been considered as an efficient method in terms of its low cost, ease of operation, simplicity, flexibility, insensitivity to toxic contaminants and has shown to be a successful alternative to traditional treatment approaches^9^. In recent years, various adsorbents have been empirically studied for their capacity for the adsorption of MG dye, such as nanoparticles^10^, nano-sheets^11^, nanocomposites^12^, polymer aerogels^13^, carbon-based materials^14^, and metal–organic frameworks (ZIF-67)^15^.

To date, a variety of applications of calcium aluminate-containing materials have been reported, including catalyst^16^, electrides and ionic conductor^17^, superhydrophobic cement^18^, biomaterial for dentistry and orthopedic fields^19^, bone cement^20^, hard tissue repair^21^, nanocatalyst for biodiesel production^22^, and adsorbent to remove aqueous Cr(VI)^23^. However, a review of the literature reveals that there are no records of monocalcium aluminate (CaAl_2_O_4_) being used to remove MG dye from aqueous media. As a result, the current research presents the first confirmation of using CaAl_2_O_4_ for MG dye adsorption from contaminated water. To obtain CaAl_2_O_4_, several synthesis methods, such as solid-state reactions^24^, sol–gel^25^, Pechini method^26^, and solution combustion method^27^ have been introduced. Sol–gel auto-combustion is an innovative approach that integrates the solution-combustion synthesis and sol–gel processes and is based on the gelling and resulting combustion of an aqueous solution consisting of an organic fuel such as citric acid and metal salts^28^. This method benefits from using low-cost precursors, simple equipment, low processing cost, high production rate, low temperature, and ultrafine particles. In this study, we describe the synthesis of meso-CaAl_2_O_4_ nanostructure using the sol–gel auto-combustion method by citric acid as a fuel.

On the other hand, different factors including the adsorbent dose, primary concentration, solution pH, temperature, and contact time have been shown to influence the efficiency of the adsorbent simultaneously^29^. As a result, optimizing the process requires a thorough understanding of how these factors interact simultaneously to impact adsorption. Thus, the experimental design as a collection of useful mathematical techniques was applied to improve the design and optimize the key parameters^30^. RSM (response surface methodology) is an effective statistical technique for simultaneously considering several independent variables and their interactions that impact the objective function. This strategy entails steadily reducing the number of trials and testing several regressions to find the conditions that yielded the best response for the methodological spectrum under consideration^31^. The central composite design (CCD) is a standard, effective, and most commonly used RSM design. In addition, in the adsorption technique, there is the serious challenge of the adsorbent regeneration with minimal efficiency loss. As a result, researchers have paid close attention to the use of several regeneration methods for reusing the adsorbent. Because of the unique molecular level heating capability of microwave-assisted regeneration technique, which leads to fast and homogeneous thermal reactions, it has recently been extensively investigated^32^.

In this study, for the first time, the citric acid-assisted sol–gel auto combustion was applied for the synthesis of meso-CaAl_2_O_4_ as adsorbent, and its structure was identified by using various state-of-the-art analytic technologies. Additionally, through utilizing CCD based on RSM, the influence of adsorption variables such as adsorbent dosage, solution pH, contact time, initial dye concentration, and solution temperature was also assessed and optimized. The equilibrium adsorption isotherm models together with kinetic models and thermodynamic of the adsorption process for fitting the experimental data were also investigated. Finally, the reusability and performance of the adsorbent in real wastewater samples were studied.

## Experimental

### Chemicals and instruments

The chemicals and equipment used in this study were all specified in the "Electronic Supplementary Information."

### Synthesis of meso-CaAl_2_O_4_

For the synthesis of meso-CaAl_2_O_4_, the citric acid-assisted sol–gel auto-combustion technique was used as follows: suitable quantities of Al(NO_3_)_3_·9H_2_O and Ca(NO_3_)_3_·4H_2_O (2:1) were dissolved in ultrapure water and kept stirring until a clear solution was obtained at room temperature. This solution was heated at 80 °C for 15 min, and citric acid was then dissolved into a minimal volume of water and added to the heated solution. The ratio of citric acid to nitrates was 1:1 (molar). The obtained reaction mixture was heated at a temperature of 80 °C, and adjusting pH to the value of 7 was carried out with the gradual addition of ammonium hydroxide solution (0.1 mol L^−1^). The mixture was gelled after 2 h of stirring. The gel was then heated for 1 h to obtain a yellowish-white mass in the oven at 200 °C and then heated at 400 °C for 30 min until the auto-combustion process took place. Finally, the calcination of the powder was carried out at 700 °C for 1 h to obtain meso-CaAl_2_O_4_.

### Adsorption procedure

**Figure 2:**
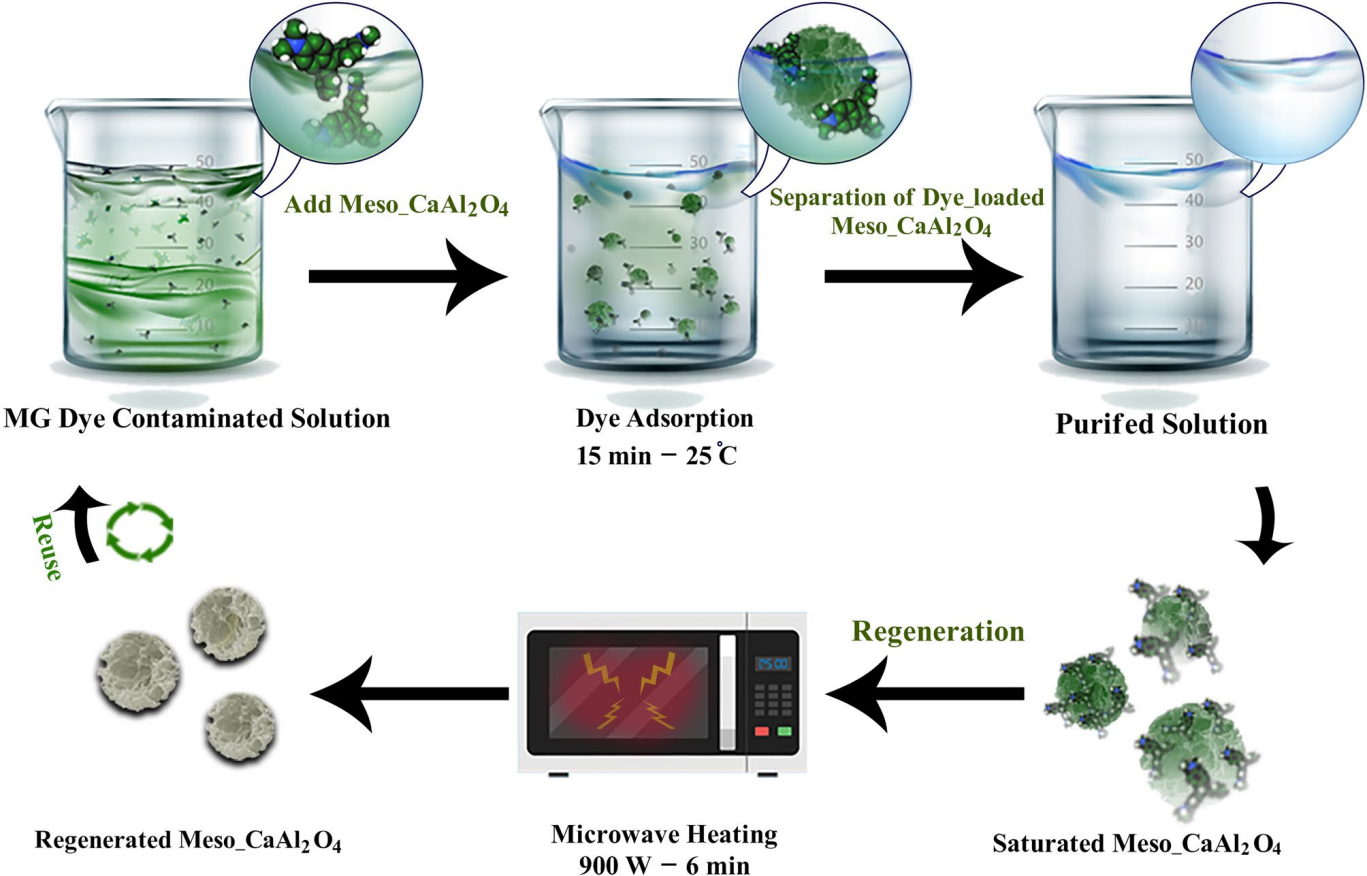
The diagram of the MG dye adsorption by meso-CaAl_2_O_4_ and microwave-assisted regeneration.

By using a shaker (digital water bath incubator Drawell Scientific, Shanghai, China), equilibrium tests were performed by the following procedure: The shaker was loaded with 50 mL of triphenylmethane dye solutions containing meso-CaAl_2_O_4_ mass (2–8 mg) and varying initial dye concentrations (20–100 mg L^−1^). Other experimental conditions were solution temperature of 5–45 °C, solution pH of 2.0–10, and contact time of 5–25 min. After performing the experiments at predetermined time intervals, the adsorbent was separated, and then the remaining MG dye in the solutions was measured spectrophotometrically at λ_max_ of 617 nm (Fig. 2).

### Central composite experimental design

CCD was performed to examine^33, 34^ the effect of contact time, solution pH, initial dye concentration, meso-CaAl_2_O_4_ mass, and solution temperature selected as the independent variables. As shown in Table 1, each factor was examined in five levels (− 2, − 1, 0, + 1, and 2) by performing 32 tests. The analysis of variance (ANOVA) statistical tests was accomplished to evaluate coefficients of determination (R^2^), and F-test was also applied to define the significance of the effect of each variable. The optimum values of the variables were attained by applying the regression equation, investigating the counter-response surface plot, and setting up constraints for the levels of the variables.

**Table 1:** CCD experimental design matrix and responses for adsorption of MG dye by meso-CaAl_2_O_4_.

Independent variables			Range and levels (coded)				
Factors	Coded	Units	− α	Low	Middle	High	+ α
pH	X_1_	–	2.0	4.0	6.0	8.0	10
MG concentration	X_2_	mg L^−1^	20	40	60	80	100
Adsorbent mass	X_3_	mg	2	3.5	5	6.5	8
Contact time	X_4_	min	5	10	15	20	25
Temperature	X_5_	°C	5	15	25	35	45

Run order	Adsorption variables					Response	
	X_1_	X_2_	X_3_	X_4_	X_5_	R% MG	
1	6.0	20.0	5.0	15	25	92.98	
2	8.0	80.0	3.5	10	35	35.76	
3	6.0	60.0	5.0	15	25	66.51	
4	6.0	60.0	5.0	15	25	63.45	
5	6.0	60.0	5.0	15	25	65.67	
6	6.0	60.0	5.0	15	25	64.33	
7	6.0	60.0	2.0	15	25	53.97	
8	8.0	40.0	6.5	10	35	98.76	
9	8.0	80.0	6.5	20	35	85.58	
10	8.0	40.0	3.5	20	35	97.45	
11	4.0	80.0	6.5	10	35	57.68	
12	8.0	40.0	3.5	10	15	68.97	
13	6.0	60.0	5.0	15	25	62.65	
14	6.0	60.0	5.0	15	45	75.88	
15	4.0	40.0	6.5	20	35	75.56	
16	6.0	60.0	5.0	5.0	25	48.93	
17	4.0	40.0	3.5	10	35	59.87	
18	6.0	60.0	8.0	15	25	91.57	
19	4.0	80.0	3.5	20	35	54.34	
20	6.0	60.0	5.0	25	25	92.56	
21	6.0	100	5.0	15	25	40.87	
22	4.0	40.0	3.5	20	15	69.87	
23	4.0	40.0	6.5	10	15	68.78	
24	4.0	80.0	6.5	20	15	70.75	
25	8.0	80.0	3.5	20	15	65.78	
26	8.0	80.0	6.5	10	15	49.87	
27	2.0	60.0	5.0	15	25	40.65	
28	6.0	60.0	5.0	15	25	65.11	
29	8.0	40.0	6.5	20	15	96.21	
30	6.0	60.0	5.0	15	5.0	54.33	
31	10	60.0	5.0	15	25	77.78	
32	4.0	80.0	3.5	10	15	8.86	

### Regeneration of adsorbent

The regeneration of the dye-loaded meso-CaAl_2_O_4_ was carried out by microwave heating method using a 2.45 GHz microwave oven with the maximum output power of 900 W in varying power levels and exposure times. After the MG dye adsorption process, the dye-loaded meso-CaAl_2_O_4_ was separated from the reaction medium and placed in the microwave oven in crucibles made of pure alumina and exposed to microwave heating. The bulk temperature of the meso-CaAl_2_O_4_ was measured by quickly inserting the thermocouple into the sample after ending the heating process with a digital display temperature controller. Finally, the regenerated meso-CaAl_2_O_4_ was washed with ultrapure water to remove the degraded MG dye on the meso-CaAl_2_O_4_ surface and was reused after drying in the oven (Fig. 2).

## Results and discussion

### Meso-CaAl_2_O_4_ characterization

**Figure 3:**
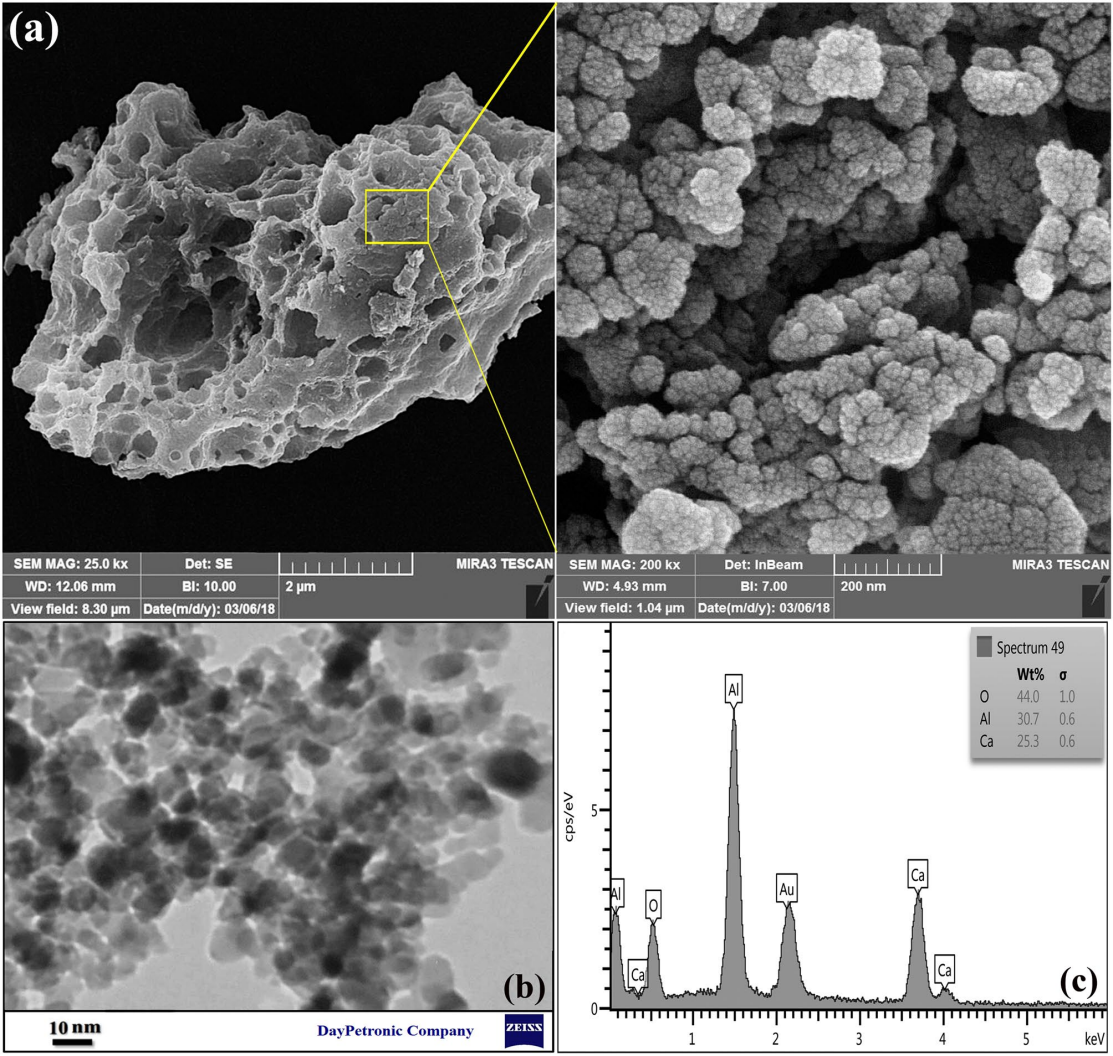
(**a**) SEM images, (**b**) TEM image, and (**c**) EDX analysis of meso-CaAl_2_O_4_.

SEM images were used to analyze the surface morphology of meso-CaAl_2_O_4_. Figure 3a shows the amorphous shape of meso-CaAl_2_O_4_ with a size of around 8 μm, which has a highly non-homogeneous surface with micrometric cavities. However, the magnified image shows that this porous structure is composed of aggregation and adhesion of near-spherical nanoparticles in the range between 10 and 20 nm. In addition, a closer look reveals that the accumulation of these nanoparticles created cavities below 50 nm in the overall structure of sample, which led to the formation of the mesoporous structure (pore size ranging from 2 to 50 nm). The formation of such a porosity in the sample results in obtaining a high value of the specific surface area in the sample. In the TEM image (Fig. 3b), this adhesion of nanoparticles of about 10 nm and the resulting holes is well visible, which is in line with SEM images. In Fig. 3c, the EDX spectrum shows the existence of Ca, Al, and O without any more peaks. It's also clear that there are no other elements in the synthesized adsorbent, and it was actually free of other impurities.

**Figure 4:**
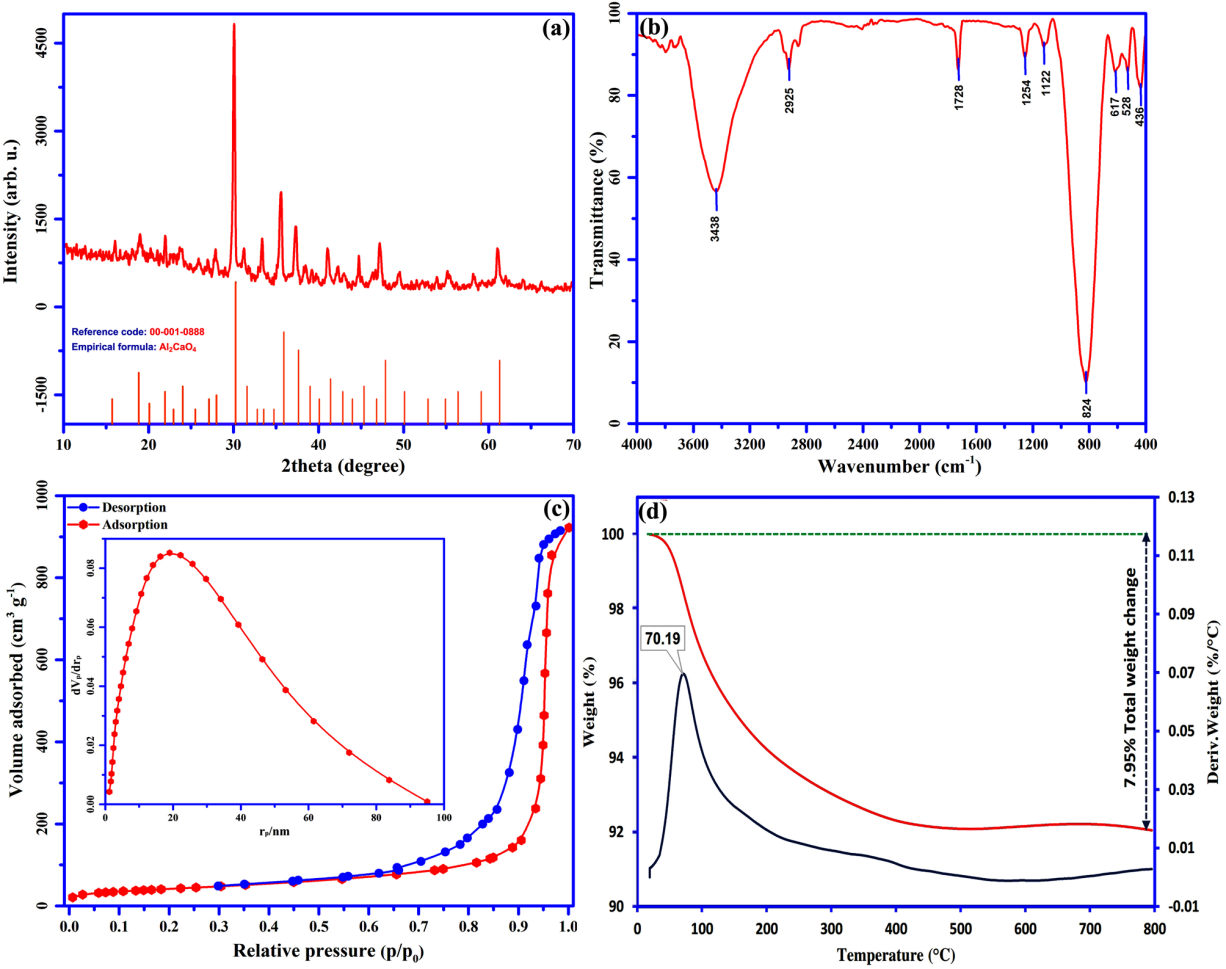
(**a**) X-ray diffraction, (**b**) FT-IR spectrum, (**c**) Nitrogen adsorption-desorption isotherm and BJH pore plot (inset), and (**d**) TG/DTG curves of meso-CaAl_2_O_4_.

The structure and phase purity of synthesized meso-CaAl_2_O_4_ was investigated by XRD and are shown in Fig. 4a. As can be seen, the successful synthesis of meso-CaAl_2_O_4_ can be proved according to JCPDS Card No. 00-001-0888, and no other impurities are found. The FT-IR spectrum was appliyed to determine the type of surface functional groups of meso-CaAl_2_O_4_ and is shown in Fig. 4b. The deformative vibration of the molecules of water is shown by stretching vibration of OH at 3437 cm^−1^ corresponding to free hydrogen-bonded hydroxyl groups^30^. The nitro groups of the precursors are responsible for other minor peaks shown at 1122 cm^−1^ and 1254 cm^−1^^35^. The stretching vibration of the M–O bond (M=Al, Ca) is seen with the strong absorption band appeared at 823 cm^−1^. The stretching vibration of the Ca–O bond is indicated at 617 cm^−1^ and 436 cm^−1^. The band at 528 cm^−1^ indicates the existence of aluminium ions confirming by the stretching vibration of Al–O. The bands at 1727 cm^−1^ and 2925 cm^−1^ are related to the existence of citrate ions that still present after calcination at 700 °C^26^. The surface area and pore structure properties of Meso-CaAl_2_O_4_ were evaluated using N_2_ adsorption/desorption isotherm, BET specific surface areas, and BJH pore size distribution analyses. According to the classification of IUPAC, the N_2_ adsorption/desorption isotherm of meso-CaAl_2_O_4_ (Fig. 4c) is a type V isotherm with a hysteresis loop of type H1, which is mostly associated with mesoporous structures. In addition, type H1 is often associated with porous materials, which consist of narrow pore-size distribution^36^. These findings mention the fact that meso-CaAl_2_O_4_ has a mesoporous structure, which is also seen in the SEM images. According to result of the BET, the adsorbent had 1.39 cm^3^ g^−1^ and 148.5 m^2^ g^−1^ of total pore volume and specific surface area, respectively. To further determine the pore size distribution of meso-CaAl_2_O_4_, the BJH equation was employed. According to IUPAC classification, pores < 2 nm are referred to as microporous, the pores ranging from 2 to 50 nm are mesoporous, and the pores > 50 nm are called macroporous^37^. Figure 4c (inset) reveals that the distribution of pores size are between 10 to 40 nm, proving the mesoporous structure of meso-CaAl_2_O_4_, and the majority of pores were around 19 nm.

Figure 4d shows the TGA-DTG curve of meso-CaAl_2_O_4_ heated from 25 to 800 °C in an air atmosphere (rate of heating = 20 °C min^–1^). According to the TGA curve, the total weight loss of the sample is 7.95%, confirming an excellent thermal resistance of meso-CaAl_2_O_4_. The major weight loss could be related to the loss of moisture in the sample, which takes place between 25 and 250 °C. After 400 °C there is no noticeable weight loss, and sample weight is constant. The main rate of weight loss is also detected by the DTG analysis at 70.8 °C, which confirms the results obtained from the TGA analysis.

### Data analysis

#### CCD model

**Figure 5:**
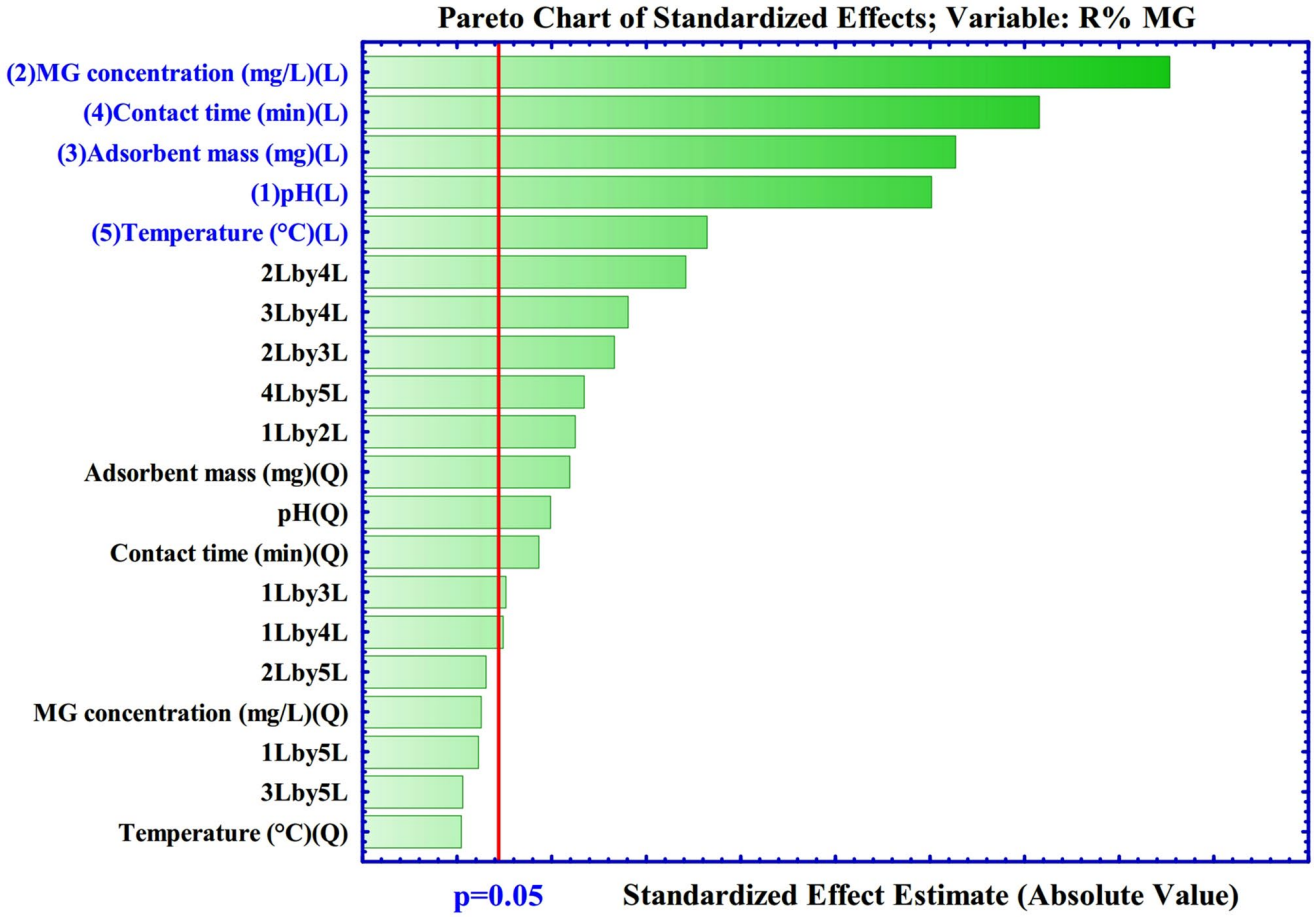
Pareto graph for the adsorption of MG dye.

For the quadratic model of MG dye adsorption, the coefficient of variation (C.V %) = 2.544% and the F-value = 217.0 (Probability > F less than 0.0001) attained by ANOVA (Table 2) imply that the model is highly significant, and the experimental results are reliable and accurate^38^. The P-value of lack of fit analysis acquired higher than 0.05 (0.2861) is not significant for the quadratic model, which approving the reliability of the model for the prediction of the MG adsorption by meso-CaAl_2_O_4_. MG concentration (X_2_), contact time (X_4_), adsorbent mass (X_3_), pH (X_1_), temperature (X_5_), the interaction effects of X_2_X_4_, X_3_X_4_, X_2_X_3,_ X_4_X_5_, X_1_X_2_, and X_1_^2^ and X_4_^2^ are significant among all terms, due to the values of the P (< 0.0001) (Fig. 5). The other coefficients are not significant (P > 0.05).

**Table 2:** ANOVA results for MG dye adsorption by meso-CaAl_2_O_4_.

Source of variation	DF	SS	MS	F-value	P-value	Status
Model	12,340	20	617.2	217.0	< 0.0001	Significant
X_1_	1784	1	1784	627.3	< 0.0001	Significant
X_2_	4032	1	4032	1418	< 0.0001	Significant
X_3_	1971	1	1971	692.9	< 0.0001	Significant
X_4_	2693	1	2693	947.0	< 0.0001	Significant
X_5_	495.1	1	495.1	174.1	< 0.0001	Significant
X_1_X_2_	110.0	1	110.0	38.67	< 0.0001	Significant
X_1_X_3_	18.86	1	18.86	6.630	0.0258	Significant
X_1_X_4_	16.67	1	16.67	5.860	0.0340	Significant
X_1_X_5_	3.540	1	3.540	1.250	0.2881	Not-significant
X_2_X_3_	195.9	1	195.9	68.89	< 0.0001	Significant
X_2_X_4_	415.9	1	415.9	146.2	< 0.0001	Significant
X_2_X_5_	6.620	1	6.620	2.330	0.1554	Not-significant
X_3_X_4_	232.3	1	232.3	81.68	< 0.0001	Significant
X_3_X_5_	0.240	1	0.240	0.090	0.7757	Not-significant
X_4_X_5_	128.1	1	128.1	45.03	< 0.0001	Significant
X_1_^2^	69.42	1	69.42	24.41	0.0004	Significant
X_2_^2^	4.440	1	4.440	1.560	0.2374	Not-significant
X_3_^2^	100.4	1	100.4	35.31	< 0.0001	Significant
X_4_^2^	52.99	1	52.99	18.63	0.0012	Significant
X_5_^2^	0.130	1	0.130	0.040	0.8362	Not-significant
Residual	31.29	11	2.840			
Lack of Fit	21.04	6	3.510	1.710	0.2861	Not-significant
Pure Error	10.25	5	2.050			
Corr Total	123,800	31				

Model summary statistics						
SD	CV%	R^2^	Adj-R^2^	Predicted	Adequate precision	
1.687	2.544	0.9975	0.9929	0.9547	67.20	

*DF* Degree of freedom, *SS* sum of squares, *MS* mean of squares.

Based on statistical results, a model for the prediction of the dye adsorption can be written as follows:

1 $${\text{R}}\%_{{{\text{MG}}}} = 30.0 + 12.55{\text{X}}_{1} - 1.80{\text{X}}_{2} + 0.82{\text{X}}_{3} + 0.79{\text{X}}_{4} + 1.04{\text{X}}_{5} - 0.07{\text{X}}_{1} {\text{X}}_{2} - 0.36{\text{X}}_{1} {\text{X}}_{3} + 0.10{\text{X}}_{1} {\text{X}}_{4} + 0.12{\text{X}}_{2} {\text{X}}_{3} + 0.05{\text{X}}_{2} {\text{X}}_{4} - 0.51{\text{X}}_{3} {\text{X}}_{4} - 0.06{\text{X}}_{4} {\text{X}}_{5} - 0.38{\text{X}}_{1}^{2} + 0.82{\text{X}}_{3}^{2} + 0.05{\text{X}}_{4}^{2}$$

The values of R^2^ = 0.998 and Adj-R^2^ = 0.993 show that a good correlation between the results gathered by Eq. (1) and experimental data exists, indicating that the values are exact and reliable. In addition, the predicted R^2^ values imply that the model has a significant block effect. The difference between the values of Adj-R^2^ (0.993) and predicted R^2^ (0.955) should be roughly 0.20^39^ and their difference shows a problem along with either the model or data. An adequate precision value of 67.20 also confirms the validity of the model.

#### Poit of zero charge and and contour plots

**Figure 6:**
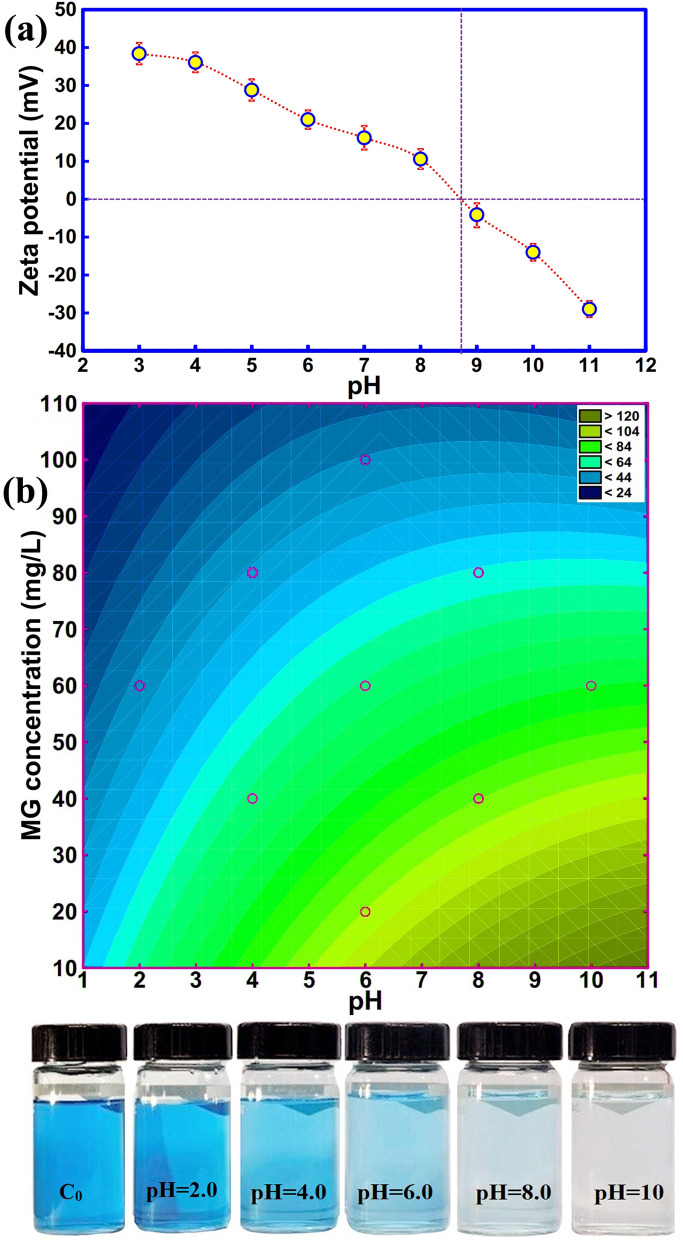
(**a**) Point of zero charge of meso-CaAl_2_O_4_ (error bars presents ± standard deviations, n = 3), and (**b**) 2D plot showing the effect of MG concentration and solution pH on the R% of MG dye.

The pH of a solution is known as a significant influencing factor that affects the adsorption performance. The point of zero charge (pHpzc) of meso-CaAl_2_O_4_ was assessed by zeta potential analysis to find its surface charge at different pH values, and according to Fig. 6a, the adsorbent is neutral, positively charged, and negatively charged at pH values = 8.7, > 8.7, < 8.7, respectively.

For a better understanding of the effect of pH, the results of the predicted models for R% MG as a function of MG concentration and pH of the solution is presented as a 2D contour plot (Fig. 6b). The corresponding figure shows the R % of MG dye at different pH values. As can be seen, in an alkaline medium, the R % is greater than in an acidic medium. By increasing pH from 2.0 to 10, the R % increases from 19.2 to 98.8%, which can be due to an enhancement in electrostatic attraction between the molecules of MG dye and meso-CaAl_2_O_4_. As shown in Fig. 6b, when an increase in the initial concentration of MG is done from 20 to 100 mg L^−1^, the percentage of adsorbed dye decreases from 98.6 to 24.9%. This may be due to the fact that higher dye concentration requires more active sites for dye adsorption. Therefore, more competition occurs between the molecules of MG dye and the binding sites of meso-CaAl_2_O_4_, and the R % of MG decreases^11^. The photographic images of MG dye adsorption by meso-CaAl_2_O_4_ at different initial pH values are presented in Fig. 6b.

**Figure 7:**
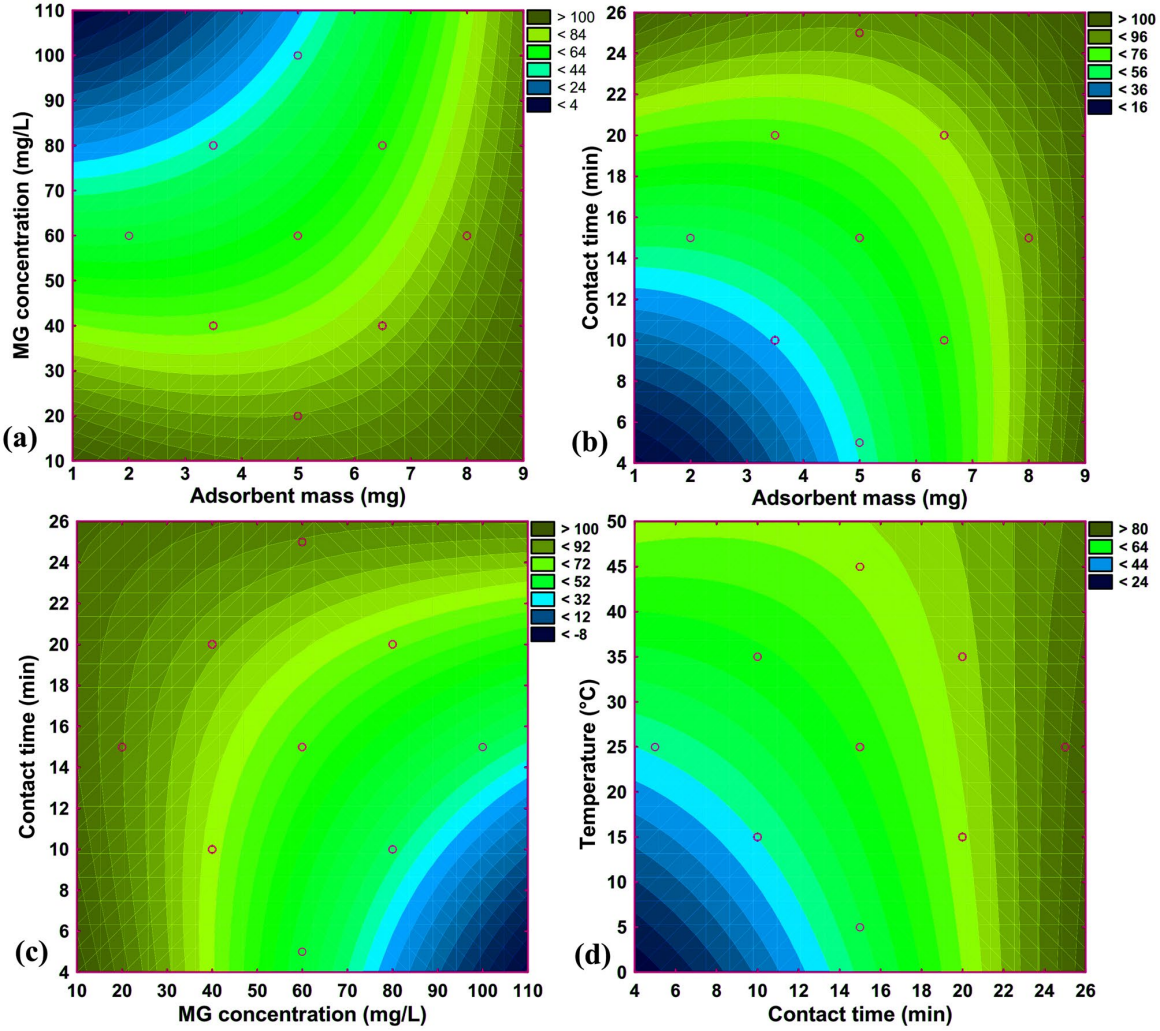
Contour plots showing the effects of (**a**) adsorbent mass-MG concentration, (**b**) adsorbent mass-contact time, (**c**) MG concentration-contact time, and (**d**) contact time–temperature on the R % of MG dye.

The simultaneous effect of the influencing parameters, including MG concentration, adsorbent mass, contact time, and temperature on the removal efficiency of MG dye by the adsorbent was assessed through a 2D contour plot and results are shown in Fig. 7.

Figure 7a shows the simultaneous effect of adsorbent mass and MG concentration on the percentage of MG dye adsorption. As can be seen, the highest R % of MG occurred in the range of the simultaneous effect of lower dye concentration and higher adsorbent mass. The adsorption percentage of MG dye increases sharply from 19.6 to 98.2%, with an enhancement in meso-CaAl_2_O_4_ mass. This may be attributed to the increased surface area and the existence of further adsorption sites. It is also a fact that increasing the adsorbent mass prevents the sites from saturating during the process^30, 40^. Figure 7b shows the simultaneous effect of adsorbent mass and contact time on the adsorption efficiency. The results showed that the removal efficiency increases with increasing time and adsorbent mass. The findings show that raising contact time from 5 to 25 min improves the R% of MG from 46.7 to 98.7%. The R % reaches 89.6% in the first 15 min of contact time. The abundance of the empty sites available on the adsorbent surface can explain the rapid adsorption of the dye at the initial stage^41^.

The adsorption performance changes almost nothing after 15 min, which could be due to the saturation of the sites of adsorption on the surface of meso-CaAl_2_O_4_ and the penetration of MG dye molecules into the pores^31, 42^. The simultaneous effect of MG concentration and contact time on the MG dye removal efficiency is shown in Fig. 7c. As shown, the highest MG dye removal efficiency was obtained at low MG concentrations and high contact times, which is related to the higher rate of active adsorption sites of the adsorbent compared to MG dye molecules at low MG concentrations. Also, increasing the contact time increases the access of dye molecules to the active adsorption sites of the adsorbent until the adsorbent reaches the saturation level. Figure 7d shows the simultaneous effect of contact time and solution temperature on the MG dye removal efficiency. The results showed that with increasing contact time and temperature, the MG dye adsorption efficiency increased. Therefore, the efficacy of MG adsorption increases significantly from 34.8 to 98.2% as the temperature rises from 5 to 45 °C. However, up to 25 °C, 96.0% of adsorption is achieved, and the impact of temperature on the adsorption performance is not significant (95% to 98.2%) at temperatures beyond 25 °C.

#### Optimal conditions for MG dye removal

**Figure 8:**
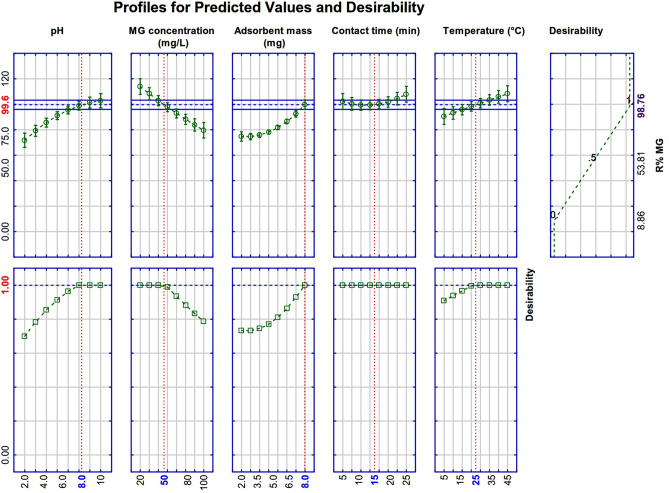
Optimal MG adsorption conditions from the model.

The optimal value of the dependent variable (R% MG) was determined by the use of the function of desirability in STATISTICA software. The experimental conditions for maximum MG removal (100%) are solution pH (X_1_) = 8.0, MG concentration (X_2_) = 50 mg L^-1^, meso-CaAl_2_O_4_ mass (X_3_) = 8 mg, contact time (X_4_) = 15 min, and solution temperature (X_5_) = 25 °C (Fig. 8). The estimated values were used in an experimental test. The experimental R% (98.68 ± 2.11%) findings were nearly identical to the value predicted by the model, indicating that the model is highly reliable.

### Adsorption isotherms modeling

The adsorption equilibrium isotherms are essential for conveying an adsorption process behavior. Four fitting models were used in this research as follows: Langmuir, Freundlich, Temkin, and Dubinin-Radushkevich (DR). Also, a dimensionless constant called separation factor (R_L_) was used to measure the nature of adsorption, in which R_L_ > 1 indicates unfavorable adsorption, R_L_ = 0 corresponds to irreversible adsorption, and 0 < R_L_ < 1 relates to favorable adsorption^43^. Table 3 lists the fitting parameters for the adsorption isotherm models at different temperatures as well as the values of correlation coefficient (R^2^) values. According to the attained values of R^2^, the Langmuir model (R^2^ = 0.999) was better fitts to other models at all temperatures, which indicates monolayer and homogeneous adsorption of MG dye molecules onto the surface of meso-CaAl_2_O_4_^44, 45^. It also reveals that the adsorbed molecules of MG dye onto meso-CaAl_2_O_4_ surfaces have no interaction^46^.

**Table 3:** Isotherm model parameters for adsorption of MG dye onto meso-CaAl_2_O_4_ (volume = 50 mL, adsorbent mass = 8 mg, contact time = 15 min, and pH 8.0).

Isotherm	Plot	Parameters	Values		
		Temperature	5 °C	25 °C	45 °C
Langmuir $\frac{{C_{e} }}{{q_{e} }} = \frac{1}{{Q_{m} k_{L} }} + \frac{{C_{e} }}{{Q_{m} }}$	*C*_*e*_*/q*_*e*_ vs. *C*_*e*_	Q_m_ (mg g^−1^)	227.0	587.5	617.1
		K_L_ (L mg^−1^)	0.247	0.235	0.293
		R^2^	0.999	0.999	0.999
		R_L_ = 1/(1 + (K_L_ × C_0_))	0.039–0.288	0.041–0.299	0.033–0254
Freundlich $lnq_{e} = lnK_{F} + \frac{1}{n}lnC_{e}$	*ln q*_*e*_ vs. *ln C*_*e*_	1/n	0.318	0.412	0.390
		K_F_ (L mg^−1^)	6.140	8.423	9.073
		R^2^	0.932	0.933	0.932
Temkin $q_{e} = B_{1} ln K_{T} + B_{1} ln C_{e}$	*q*_*e*_ vs. *ln C*_*e*_	B_1_	40.361	115.6	115.1
		K_T_ (L mg^−1^)	1.000	1.000	1.000
		R^2^	0.972	0.985	0.988
Dubinin–Radushkevich (D–R) $ln q_{e} = lnQ_{s} - k\varepsilon^{2}$	*ln q*_*e*_ vs. *ε*^*2*^	Q_S_ (mg g^−1^)	183.6	425.5	444.3
		β × 10^–7^	4.189	3.473	2.129
		E (kJ mol^−1^)	1.092	1.200	1.533
		R^2^	0.796	0.813	0.795

The results suggest that the Langmuir theoretical maximum monolayer adsorption capacity increases from 227.0 to 617.1 mg g^–1^ with an increase in temperature from 5 to 45 ºC. However, the rate of increase in adsorption capacity is not significant with increasing temperature from 25 to 45 °C, and the maximum monolayer adsorption capacity of 587.5 mg g^–1^ obtained at 25 °C is comparable to several other adsorbents applied in recent years to remove MG dye from aqueous medium (Table 4). Hence, the characteristics and performance of the investigated adsorbent, meso-CaAl_2_O_4_, support its appropriate as one potential adsorbent for removal of cationic pollutants such as MG from polluted aqueous systems. In addition, the R_L_ values (0 < R_L_ < 1), confirming favorable adsorption of MG dye onto meso-CaAl_2_O_4_^47^. In addition, The value of energy of adsorption (E) obtained from the D-R isotherm model (below 8 kJ mol^−1^ in all temperatures) suggests physical adsorption can be considered effective in the adsorption process^12^.

**Table 4:** Comparison of meso-CaAl_2_O_4_ with other materials reported in the literature for MG dye adsorption (estimated by Langmuir isotherm).

Adsorbent	Q_max_ (mg g^−1^)	Ref.
Super paramagnetic sodium alginate-coated Fe_3_O_4_-NPs	47.84	^48^
Graphene oxide/cellulose bead composites	30.09	^49^
Montmorillonite clay	262.5	^50^
Starch-graft-poly(acrylamide)/GO/hydroxyapatite nanocomposite	297.0	^51^
Fe_3_O_4_@SiO_2_-GO	265.9	^52^
3D magnetic bacterial cellulose nanofiber/GO polymer aerogel	270.3	^13^
Hematite-reduced graphene oxide composites	438.8	^53^
Nano-iron oxide-loaded alginate microspheres	2.30	^54^
Tin oxide nanoparticle loaded on activated carbon	142.9	^55^
Gold nanoparticles loaded on activated carbon	164.6	^56^
NiS nanoparticles synthesized using plant leaf extract	64.85	^57^
TiO_2_ Nanoparticles	6.30	^58^
Activated carbon/CoFe_2_O_4_ composite	89.29	^59^
Graphene oxide and reduced graphene oxide	13.52	^60^
Zeolite nanostructures	226.8	^61^
Expanded graphite	41.49	^62^
Reduced graphene oxide	476.2	^11^
Mesoporous exfoliated graphite	384.6	^63^
Amino functionalized graphenes	91.48	^64^
Activated carbon derived from biomass	48.48	^65^
Functionalized multi walled carbon nanotubes	142.9	^66^
Bentonite	178.6	^67^
Organo clay	56.82	^68^
Natural zeolite	36.50	^69^
Meso-CaAl_2_O_4_	587.5	This work

### Adsorption kinetics modeling

The kinetics of MG dye adsorption by meso-CaAl_2_O_4_ were investigated using pseudo-first-order, pseudo-second-order, intra-particle diffusion, and Elovich kinetic models under different solution pH conditions, and results are given in Table 5. As can be seen, the pseudo-second-order kinetic model fitted well for the whole range of solution pH conditions, showing the best correlation coefficient (R^2^) values. This implies that the process of MG dye adsorption in a wide range of solution pH (2–10) mostly follows chemical adsorption^70^. It should be noted that the best (R^2^) values are obtained at a solution pH of 8 (R^2^ = 0.999), which indicates that the adsorption is more appropriate at this pH. Furthermore, the qe value calculated from the pseudo-second-order model (q_e_, calc) and the experimental qe (q_e_, exp) value are very close to each other, emphasizing the suitability of the pseudo-second-order model.

**Table 5:** Kinetic model parameters and correlation coefficients for adsorption of MG dye onto meso-CaAl_2_O_4_ (volume, 50 mL; initial concentration, 50 mg L^-1^; adsorbent dose, 8 mg; solution temperature, 25 ºC).

Model	Plot	Parameters	Values				
		pH	2.0	4.0	6.0	8.0	10
First-order- kinetic $\ln (q_{e} - q_{t} ) = \ln q_{e} - k_{1} t$	*ln* (*q*_*e*_* − q*_*t*_) vs. *t*	k_1_ (min^−1^)	0.257	0.251	0.251	0.301	0.301
		q_e (calc)_ (mg g^−1^)	318.4	411.0	519.9	727.8	746.1
		R^2^	0.770	0.719	0.715	0.944	0.920
Pseudo-second-order-kinetic $\frac{t}{{q_{t} }} = \frac{1}{{k_{2} q_{e}^{2} }} + \frac{t}{{q_{e} }}$	*t/q*_*t*_ vs.* t*	k_2_ (min^−1^)	0.0006	0.0003	0.0003	0.0002	0.0002
		q_e (calc)_ (mg g^−1^)	252.8	373.0	457.2	595.3	618.1
		R^2^	0.992	0.996	0.992	0.999	0.992
		h (mg g^−1^ min^−1^)	37.33	40.77	54.17	73.4	70.36
Intraparticle diffusion $q_{t} = k_{diff} t^{1/2} + C$	*q*_*t*_ vs.* t*^*1/2*^	K_diff_ (mg g^-1^ min^−1/2^)	45.40	64.39	80.42	105.4	107.94
		C (mg g^−1^)	0.75	16.85	17.76	18.66	25.72
		R^2^	0.990	0.990	0.992	0.973	0.977
Elovich $q_{t} = \frac{1}{\beta }\ln (t) + \frac{1}{\beta }\ln (\alpha \beta )$	*q*_*t*_ vs.* ln t*	β (g mg^−1^)	0.019	0.013	0.011	0.008	0.008
		α (mg g^−1^ min^−1^)	87.38	100.85	131.22	174.9	171.04
		R^2^	0.984	0.985	0.978	0.984	0.982
Experimental data		q_e (exp)_ (mg g^−1^)	185.91	245.42	310.86	397.31	400.51

### Thermodynamics of the adsorption

Van't Hoffs plot [ln K_C_ vs 1/T(K)] was used to quantify thermodynamic parameters, such as Gibbs free energy change (∆G°), entropy change (∆S°), enthalpy change (∆H°), and explain the thermodynamic behavior of MG dye adsorption onto meso-CaAl_2_O_4_. Thermodynamic parameters of the MG dye adsorption onto meso-CaAl_2_O_4_ at different MG dye concentrations are presented in Table 6. As can be seen, the ∆G° values are in a negative range that implies the spontaneous MG dye adsorption onto meso-CaAl_2_O_4_^71^. Also, the ∆G° values decrease as the temperature rises, meaning that adsorption seems to be more desirable at higher temperatures. The obtained positive quantity of ∆H° (45.68, 47.15, and 11.11 kJ mol^–1^) indicates that the process of MG dye adsorption is done endothermically^72^. In MG concentrations of 25 and 50 mg L^−1^, a significant interaction between the MG dye and meso-CaAl_2_O_4_ is also concluded by the high value of ∆H°^73^. However, decreasing the ∆H° at higher MG concentrations (100 mg L^−1^) indicates a decrease in the interactions between the meso-CaAl_2_O_4_ and the MG dye, which can be due to the quick saturation of active sites on the exterior surface of meso-CaAl_2_O_4_ at higher concentrations and prevents the accessing of more dye molecules to more unoccupied active sites. The values of ∆S° (181.55, 183.6, and 39.05 J mol^–1^,) are also positive, which suggests the increment of randomness at the interface of the meso-CaAl_2_O_4_/solution during the process of MG dye adsorption^74^. This degree of randomness has decreased with decreases the mobility of molecules at higher MG concentrations (100 mg L^−1^).

**Table 6:** Thermodynamic parameters for the adsorption of MG dye onto meso-CaAl_2_O_4_ (volume = 50 mL, adsorbent mass = 8 mg, contact time = 15 min, and solution pH 8.0).

MG concentration	25 mg L^−1^		50 mg L^−1^		100 mg L^−1^	
T(k)	k_C_	ΔG° (kJ mol^−1^)	k_C_	ΔG° (kJ mol^−1^)	k_C_	ΔG° (kJ mol^−1^)
278.15	8.185	− 4.86	5.337	− 3.87	0.887	0.28
288.15	13.78	− 6.28	9.194	− 5.31	1.021	− 0.05
298.15	34.89	− 8.80	28.03	− 8.26	1.319	− 0.69
308.15	56.31	− 10.33	38.54	− 9.35	1.461	− 0.97
318.15	100.3	− 12.19	80.08	− 11.59	1.607	− 1.25
328.15	150.9	− 13.69	103.6	− 12.66	1.838	− 1.66
R^2^	0.992		0.978		0.983	
ΔS° (J mol^-−1^ k^−1^)	181.55		183.6		39.05	
ΔH° (kJ mol^-1^)	45.68		47.15		11.11	

### Proposed adsorption mechanism

The MG dye molecules can be adsorbed onto meso-CaAl_2_O_4_ via different mechanisms such as electrostatic interactions, pore diffusion mechanism, hydrogen bonding, and chemical bonding. One of the important experimental data used for the investigation of adsorption mechanisms is those obtained from adsorption efficiency at various values of solution pH. The solution pH affects the dissociation constant (pK_a_) of MG dye and the pHpzc of meso-CaAl_2_O_4_. As shown in Fig. 6a, meso-CaAl_2_O_4_ has a positive or negative surface charge in different solution pH conditions. In addition, the dye molecules also have a cationic or anionic nature at different values of solution pH. Therefore, under different solution pH conditions, the meso-CaAl_2_O and the MG dye molecules are different in electric charge and can interact through electrostatic interactions. The change in the percentage of adsorption under different solution pH conditions is evidence that electrostatic interactions has been one of the influential factors in adsorption. The presence of the pore diffusion mechanism is also possible owing to the high surface area and extremely porous nature of meso-CaAl_2_O_4_. As a result of pore diffusion or capillary condensation, the molecules of MG can be adsorbed by diffusing into the pores of meso-CaAl_2_O_4_^75^.

**Figure 9:**
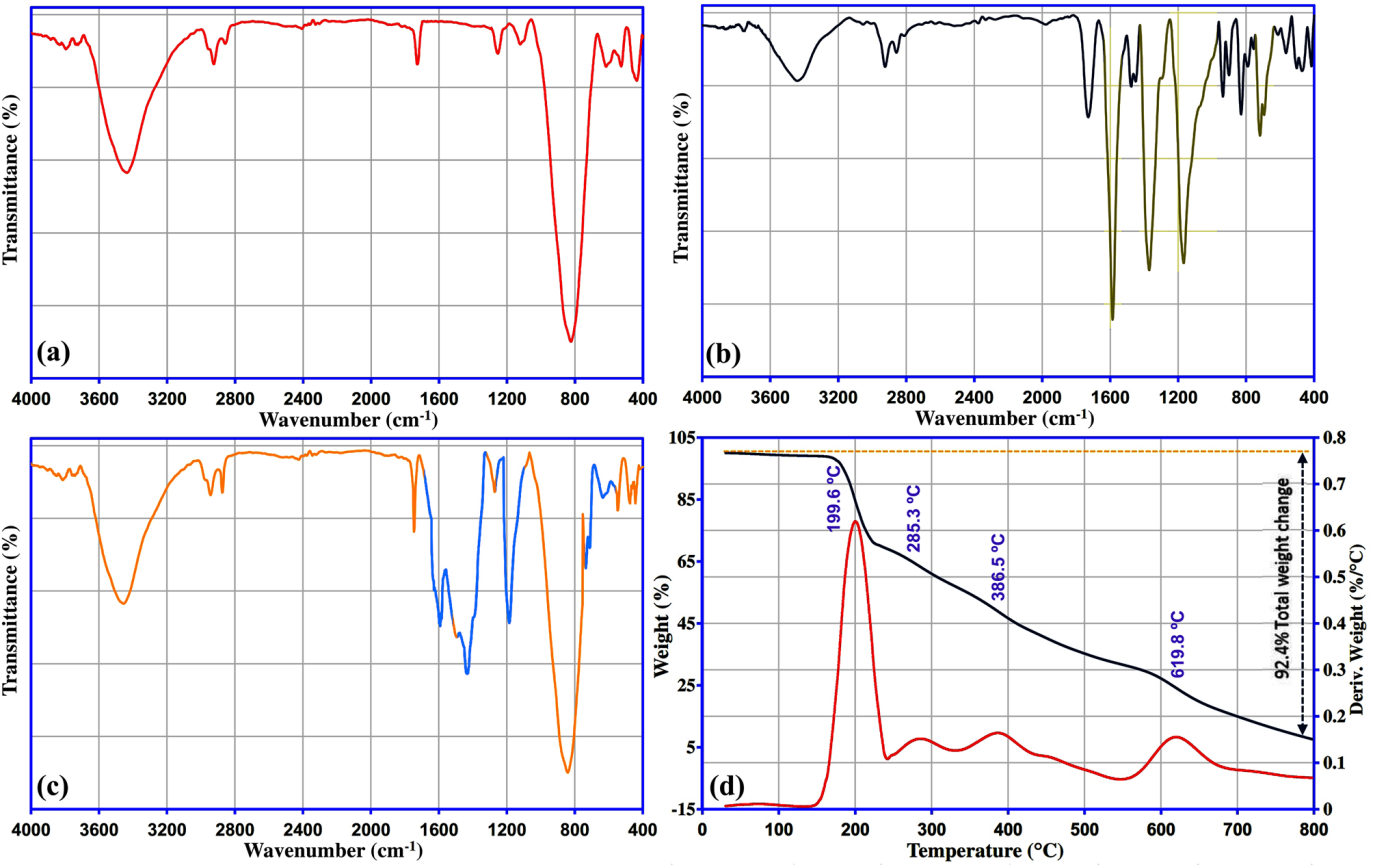
FT-IR spectra of (**a**) meso-CaAl_2_O_4_ before adsorption of MG dye, (**b**) MG dye, (**c**) meso-CaAl_2_O_4_ after adsorption of MG dye, and (**d**) TGA/DTG curves of MG dye.

**Figure 10:**
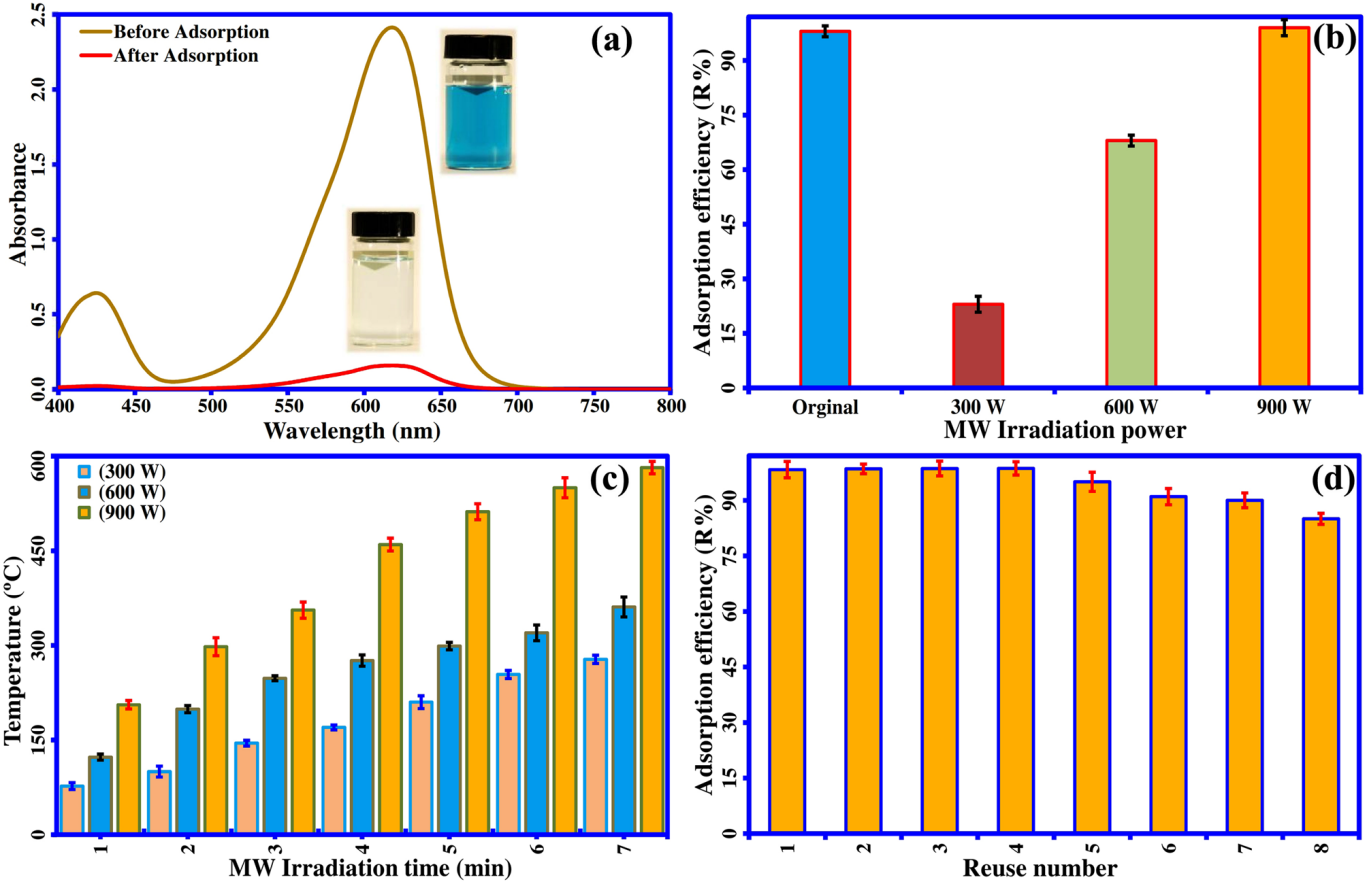
(**a**) UV–Vis spectra of MG dye solution before and after adsorption (adsorbent mass = 8 mg, V = 50 mL, T = 25 °C, pH 8.0, and contact time = 15 min), (**b**) the R% of regenerated adsorbent irradiated at various MW powers, (**c**) the temperature profiles of the sample measured by the thermocouple at different MW powers, and (**d**) the R% of MG dye onto meso-CaAl_2_O_4_ at different cycles (error bars presents ± standard deviations, n = 3).

Furthermore, the probability of chemical bond participation in the adsorption process is illustrated by analyzing the surface functional groups of meso-CaAl_2_O_4_ before and after the MG dye adsorption. As shown in the FT-IR spectra (Fig. 9), after the MG dye adsorption onto meso-CaAl_2_O_4_, several characteristic peaks of MG (indicated with different colors) are found in the meso-CaAl_2_O_4_ spectrum, which has not appeared in the spectrum of blank meso-CaAl_2_O_4_. This implies that adsorption probably also occurred through chemical bonds. This fact is also evident from the results of the adsorption kinetic assessment, in which the pseudo-second-order fitting model describes the process of MG adsorption. Further study of the FT-IR results reveals that the absorption peaks corresponding to oxygen-containing stretching vibrations on the meso-CaAl_2_O_4_ were shifted after MG adsorption, implying that the hydrogen bonding is formed between meso-CaAl_2_O_4_ and MG dye molecules during the adsorption process. Finally, MG adsorption by meso-CaAl_2_O_4_ was also confirmed by the UV–Vis spectroscopy results (Fig. 10a). It can be implied that the MG absorbance value decreases at 617 nm after the adsorption process. These results confirm the successful adsorption of MG onto meso-CaAl_2_O_4_. The photographic images of the MG solution before and after its adsorption onto the adsorbent are presented in Fig. 10a.

### Application of the adsorbent for the treatment of industrial effluent

The MG dye adsorption investigation for real applications was performed at optimal conditions (adsorbent mass = 8 mg, contact time = 15 min, initial MG dye concentration = 50 mg L^−1^, solution pH of 8.0, and solution temperature of 25 °C). The mean percentage of MG removal from a real textile effluent is shown in Table 7. According to the obtained values, the efficiency of the adsorbent for MG adsorption is 72.12% ± 1.81, which indicates the effectiveness of meso-CaAl_2_O_4_ for real applications. Also, the characteristics of the textile wastewater before and after treatment are given in Table 8.

**Table 7:** Data of the precision for the adsorption of MG dye onto meso-CaAl_2_O_4_ in the textile wastewater (volume = 50 mL, initial dye concentration = 50 mg L^−1^, adsorbent mass = 15 mg, contact time = 15 min, T = 25 °C, pH 8.0).

Number	Within-day (repeatability)	Between-day (reproducibility)
1	70.85	70.56
2	73.24	71.23
3	71.90	73.45
4	75.80	71.76
5	69.45	72.97
Mean ± SD	72.12 ± 1.81

**Table 8:** Characterization of the textile wastewater before and after treatment.

Characteristic	Unit	Treatment		Reduction %
		Before	After	
Temperature	C	26.8	27.1	–
Conductivity	µS cm^−1^	2985	869	70.9
Total dissolved solids (TDS)	mg L^−1^	1910	493	74.2
Chemical oxygen demand (COD)	mg L^−1^	1262	130	89.7
Biochemical oxygen demand (BOD_5_)	mg L^−1^	336	41	87.8
Total suspended solids (TSS)	mg L^−1^	349	92	73.6
Color	Pt–Co	1360	51	96.2
Turbidity	NTU	136	3.3	97.6
pH	–	8.1	7.9	–

### Microwave-assisted regeneration of the adsorbent

The regeneration of adsorbent is a critical factor for its application to be the effectiveness and economical. Owing to this reason, achieving a faster and easier regeneration method has always been considered. In this regard, the microwave-assisted regeneration of dye-loaded meso-CaAl_2_O_4_ was studied. Microwave power in the microwave heating method plays an essential role in the amount of temperature produced and can help regenerate the adsorbent. The changes in the temperature of meso-CaAl_2_O_4_ at different microwave powers (300 W, 600 W, and 900 W) are shown in Fig. 10c. It is seen that an increase in temperature is obtained by a rise in the power of the microwave. In addition, the adsorption efficiencies of the regenerated meso-CaAl_2_O_4_ at various microwave powers are shown in Fig. 10b. The results express that the R % of MG is greater with meso-CaAl_2_O_4_ treated at higher microwave power. This can be due to the higher temperature produced at higher powers, which results in the higher degradation of MG from meso-CaAl_2_O_4_.

In order to investigate and confirm MG degradation by microwave heating process, the TGA/DTG was applied. The results in Fig. 9d show that degrading MG begins at the temperature of around 200 °C, and at 800 °C, it loses around 92.4% of its mass. However, the adsorbent shows a very high heat resistance, and it loses only 7.95% of its weight up to 800 °C, which is mostly corresponding to the moisture as mentioned before (Fig. 4d). Therefore, the results confirm a successful regeneration of meso-CaAl_2_O_4_ using the microwave-assisted heating method. Finally, the regeneration of the dye-loaded meso-CaAl_2_O_4_ was studied using the microwave-assisted heating method. For this purpose, an evaporating dish containing the saturated adsorbent was placed into the microwave and treated for 6 min at 900 W. After the heating process, the treated meso-CaAl_2_O_4_ was washed three times with ultrapure water to thoroughly eliminate the carbon black and other materials left over from the MG degradation. The washed adsorbent was then placed in an oven at 110 °C to be dried for further use. The recyclability of meso-CaAl_2_O_4_ treated with microwave heating method is shown in Fig. 10d. As seen from the results, the adsorbent has a good reusability, so that after five reuses, the adsorption percentage of MG is still > 90%, which indicates that the adsorbent is efficient and economical.

## Conclusions

A citric acid-assisted sol–gel auto-combustion process was used to successfully synthesize meso-CaAl_2_O_4_ as an adsorbent, which was then utilized for efficient removal of MG, a cationic dye, from synthetic/real effluent. The synthesized adsorbent was characterized using different techniques. The values obtained for specific surface area, BJH pore diameter, and total pore volume of meso-CaAl_2_O_4_ were 148.46 m^2^ g^−1^, 19 nm, and 1.39 cm^3^ g^−1^, respectively. The adsorption optimal conditions, solution pH (X_1_) = 8.0, MG concentration (X_2_) = 50 mg L^−1^, meso-CaAl_2_O_4_ mass (X_3_) = 8 mg, contact time (X_4_) = 15 min and solution temperature (X_5_) = 25 °C, was obtained based on RSM-CCD. Langmuir isotherm fitting model well explained the equilibrium adsorption of MG onto the adsorbent, presenting 587.5 mg g^−1^ as the maximum monolayer adsorption capacity. Pseudo-second-order kinetic model carried out very well the fitting of the experimental kinetic data. Furthermore, the attained values of thermodynamic parameters (positive values of ∆H° and ∆S°, and negative values of ∆G°) showed the adsorption process of MG dye to be endothermic and spontaneous, and suggested the increment of randomness at the interface of the meso-CaAl_2_O_4_/solution during the process of MG adsorption. The dye-loaded meso-CaAl_2_O_4_ was successfully regenerated by the microwave-assisted heating method, and the adsorption percentage of MG dye was still > 90% after five reuses. In conclusion, the meso-CaAl_2_O_4_ can be suggested for the efficient removal of hazardous dyes, especially MG dye, from wastewaters.

## Supplementary Information

Supplementary Information.
