# Supplementary material for: A reusable mesoporous adsorbent for efficient treatment of hazardous triphenylmethane dye wastewater: RSM-CCD optimization and rapid microwave-assisted regeneration
Source: Sci Rep. 2021 Nov 23;11:22751. doi: 10.1038/s41598-021-02213-2 (PMC8610993; doi:10.1038/s41598-021-02213-2)
Supplement: Supplementary file 1 — Supplementary Information. [file 41598_2021_2213_MOESM1_ESM.doc]

**Supporting Information**

**A reusable mesoporous adsorbent for efficient treatment of hazardous triphenylmethane dye wastewater: RSM-CCD optimization and rapid microwave-assisted regeneration**

Payam Arabkhani **^a^**, Hamedreza Javadian ^b^, Arash Asfaram ^c,*^, Seyed Nabiollah Hosseini ^a^

^a^ Department of Chemistry, Tehran North Branch, Islamic Azad University, Tehran, Iran

^b^ Chemistry & Chemical Engineering Research Center of Iran (CCERCI), P.O. Box 14335-186, Tehran, Iran

^c^ Medicinal Plants Research Center, Yasuj University of Medical Sciences, Yasuj, Iran

***^*^Corresponding author.***

*Email address: arash.asfaram@yums.ac.ir*

**Section S.2.1.**

All chemicals in this work were of analytical grade and were used without further purification. Aluminum nitrate (Al(NO_3_)_3_·9H_2_O) and calcium nitrate (Ca(NO_3_)_3_·4H_2_O) were purchased from Merck (Darmstadt, Germany) and used as the metallic precursors. The citric acid (C_6_H_8_O_7_ H_2_O) and ammonium hydroxide (NH_4_OH, 25% of NH_3_) were purchased from Sigma–Aldrich (Steinheim, Germany) and used as the sacrificial organic fuel and adjust pH at 7 to the formation of gel, respectively. Sodium hydroxide (NaOH) and hydrochloric acid (HCl) were purchased from Merck (Darmstadt, Germany) and used to adjust the pH to the acidic or alkaline environment in adsorption experiments. The MG dye with the molecular formula of C_23_H_25_N_2_ (MW=329.46, λ_max_= 617 nm) was purchased from Sigma–Aldrich (M) (Sdn Bhd, Malaysia) and was dissolved in ultrapure water to prepare the stock solution for adsorption study. The ultrapure water was produced by a Milli-Q system (Millipore, Bedford, MA, USA).

Structural analysis and phase purity of meso-CaAl_2_O_4_ was performed using X-ray diffraction (XRD, Philips, X'Pert MPD, Netherlands, 40 kV, and 30 mA) was recorded over 2θ values ranging from 10 to 70º using Cu Kα radiation (λ=1.5406 Å). The Fourier transform infrared spectra (FT-IR, Thermo Nicolet, Avatar 360, USA) applied to determine the functional groups of the sample over the wavenumber range of 400−4000 cm^−1^. Scanning electronic microscopy (SEM, TESCAN, MIRA 3XMU, Czech Republic) equipped with energy dispersive X-ray spectroscopy (EDX) was used to investigate the surface morphologies, particle size, and elemental analysis of the sample. The thermal behavior was investigated using thermogravimetric analysis (TGA) and derivative thermogravimetric analysis (DTG) was carried out in a thermal analysis system (TA, Q-600, USA). The sample was heated at a temperature range from 25 to 1000 ºC with a constant rate of 10 ºC/min, in air atmosphere. The specific surface area (m^2^ g^−1^), total pore volume (cm^3^ g^−1^) and mean pore diameter (nm) of the sample were measured using nitrogen adsorption/desorption isotherms with a Brunauer–Emmett–Teller (BET) analysis (BEL, Belsorp- mini II. Japan) and measured at 77 K. The sample was degassed to remove physically absorbed gases from the sample surface, in particular, water vapor at 120 ºC for 2 h before nitrogen adsorption measurements. The concentration of MG dye was determined by UV-VIS spectrophotometer (UV-Vis, HACH, DR 5000, USA) at 617 nm. The dye aqueous solution pH was measured by a pH meter (Metrohm, Herisau, model 692, Switzerland).
